# Gender disparities in heart failure diagnosis and management: under the radar

**DOI:** 10.3389/fcvm.2026.1893614

**Published:** 2026-07-29

**Authors:** Irina Cabac-Pogorevici, Iuliana Creangă, Inessa Jitari, Dmitri Savca, Valentin Avram, Alexandru Corlăteanu, Valeriu Revenco

**Affiliations:** 1 Cardiology Laboratory, “Nicolae Testemițanu” State University of Medicine and Pharmacy, Chișinău, Republic of Moldova; 2 Discipline of Cardiology, “Nicolae Testemițanu” State University of Medicine and Pharmacy, Chișinău, Republic of Moldova; 3 Discipline of Pneumology and Allergology, “Nicolae Testemițanu” State University of Medicine and Pharmacy, Chișinău, Republic of Moldova

**Keywords:** biomarkers, gender disparities, guideline-directed medical therapy, heart failure, sex differences

## Abstract

Heart failure (HF) remains a leading cause of morbidity and mortality worldwide, affecting women and men in nearly equal numbers, yet important sex- and gender-related differences remain insufficiently recognized in clinical practice and research. Biological sex and gender influences profoundly the disease development, clinical presentation, diagnosis and management. For decades, most evidence and guideline recommendations have been based on predominantly male cohorts, limiting their applicability to women. This review aims to synthesize the current evidence on sex- and gender-related differences in HF. Also, it integrates data from experimental studies, clinical trials, large registries, and recent guidelines, emphasizing differences in phenotype, biomarker profiles, pharmacologic responses, and access to device-based therapies. Despite better survival, women report greater symptom burden, poorer quality of life, and lower use of optimal medical or device therapy, including lower use of guideline-directed medical therapy and device-based interventions. Pharmacokinetic and pharmacodynamic differences suggest that women may achieve comparable therapeutic benefit at lower medication doses, yet guidelines remain largely sex-neutral. Thus, recognizing these biological and sociocultural distinctions is essential for precision and equity in care, improving diagnostic accuracy, optimizing therapeutic strategies and ensuring equity in cardiovascular care, advancing truly individualized cardiovascular medicine.

## Introduction

### Reframing heart failure through a gender Lens

Heart failure (HF) represents a leading health threat for both women and men, particularly the elderly (1). However, evidence shows that the experience, diagnosis, treatment, and outcomes of HF substantially differ between sexes. Historically, cardiovascular research and guideline development have been grounded in male-dominant population, especially, in heart failure with reduced ejection fraction (HFrEF), leading to limited external validity for women (2, 3). As a matter of fact, a review of 740 cardiovascular studies revealed that women accounted for only 38.2% of participants overall and 23.1% in pharmacologic HF trials (2). In contrast, real-world registries such as CHECK-HF report a higher proportion of women (33.6%) among patients with HFrEF, underscoring a gap between trial representation and clinical reality (2, 4).

Growing recognition of sex-specific biological and pharmacokinetic differences—spanning myocardial structure, hormonal modulation, and neurohormonal activation—has begun to shift this paradigm (1, 5). Reframing HF through a gender lens is critical for developing equitable evidence-based diagnostic and therapeutic strategies.

For the purpose of this review, “sex” refers to biological attributes determined by chromosomal complement, reproductive organs, hormonal milieu, and physiological characteristics that distinguish females and males. In contrast, “gender” refers to the socially constructed roles, behaviors, identities, and norms that influence health-related behaviors, healthcare access, and clinical outcomes. Accordingly, sex differences discussed in this review primarily reflect biological mechanisms, whereas gender differences refer to sociocultural factors that affect disease recognition, management, and prognosis (1, 3, 5).

### The overlooked role of sex and gender in cardiovascular care

Current HF guidelines still lack sex-specific therapeutic recommendations, despite growing evidence that women may achieve comparable prognostic outcomes at lower doses of standard HF medications than men receiving full guideline-directed therapy (2, 5). Beyond biological differences, gender as a social construct significantly shapes how symptoms are perceived, reported, and managed. Gender-related behavioral patterns, health-seeking attitudes, and patient–clinician interactions all influence disease recognition and care delivery (1, 3). Women frequently present with atypical or non-specific manifestations—particularly in heart failure with preserved ejection fraction (HFpEF)—leading to diagnostic delays or misclassification. Moreover, sociocultural factors, unequal access to care, and implicit provider bias contribute further to the persistent disparities in treatment and outcomes between women and men (1, 3, 5).

### From under-recognition to undertreatment: a persistent gap

Despite well-established clinical and pathophysiological differences, current clinical practice often approaches HF as a homogeneous condition overlooking sex- and gender-specific characteristics. Women remain less likely to receive guideline-directed medical therapy (GDMT), device referrals, or inclusion in clinical trials, even though available evidence indicates they may respond differently to treatment (2, 3).

Adverse drug reactions are reported more frequently and tend to be more severe in women, adding further complexity to pharmacologic management (2). Beyond biological factors, gender-related determinants including emotional well-being, social support, healthcare access, and caregiving roles influence significantly disease experience and outcomes. These sociocultural dynamics shape how women perceive symptoms, seek care, and engage with the health system (1, 5).

Together, these findings reveal a consistent pattern of under-recognition and undertreatment of women with HF, both in research and clinical practice, underscoring the need for sex- and gender-sensitive approaches to management.

## The aim and scope of the review

### The objective of the review

To synthesize current evidence on sex- and gender-related disparities across the HF continuum—spanning epidemiology and phenotypes (HFpEF vs. HFrEF)**,** diagnostic evaluation (clinical presentation, biomarkers, imaging), and management (GDMT, devices, and advanced therapies)—and to highlight implications for practice, guideline development, and research design**.**

### Rationale for focusing on sex and gender related disparities in HF

Although HF affects women and men in comparable proportions, women remain consistently under-recognized, under-diagnosed, and under-treated. Biological differences including variations in cardiac structure, vascular and neurohormonal regulation, metabolism, and pharmacokinetics intersect with gender-related factors such as symptom perception, healthcare access, and clinician bias. These interactions shape distinct clinical phenotypes, diagnostic pathways, therapeutic responses, and quality-of-life outcomes. Addressing these disparities is essential for:

- enhance diagnostic evaluation through better recognition of sex-specific clinical patterns;
- optimize dose selection and treatment strategies within GDMT;
- promote equitable access to devices, advanced therapies, and follow-up care;
- and strengthen research design by ensuring balanced representation and sex-stratified data analysis.

### Overview of methodology used to select and synthesize the literature (narrative approach)

This is a narrative review integrating mechanistic, clinical, and health-services literature. We prioritized:

- Peer-reviewed studies, major randomized trials, registries, guidelines/consensus statements, and high-quality reviews relevant to sex/gender in HF;
- Evidence addressing diagnostic presentation (clinical signs, biomarkers, imaging), treatment response and tolerability (GDMT classes, device/advanced therapies), and outcomes (mortality, hospitalization, health status/quality of life);
- Studies reporting sex-stratified data or enabling inference about sex/gender effects.

Given heterogeneity across designs and endpoints, we synthesized themes qualitatively rather than performing a meta-analysis. Pediatric/congenital HF and non-cardiac conditions were excluded unless directly informing adult HF sex differences. Where data were conflicting or limited, we explicitly note knowledge gaps and future research needs.

## Sex-based biological differences in heart failure

### Diverging trajectories: sex-specific patters in heart failure phenotypes

#### HFpEF and HErEF: contrasting gender distributions

HF exhibits distinct phenotypic patterns across sexes. Besides, HFpEF predominates among women, while HFrEF is more prevalent in men (6, 7). Data from the Framingham Heart Study revealed a declining incidence of HFrEF and a concurrent rise in HFpEF over the past decades, reflecting demographic aging and the disproportionate burden in women (7).

Women with HFpEF are typically older, more often hypertensive and obese, and less likely to have coronary artery disease or atrial fibrillation than men (8). Clinically, they experience more congestion symptoms and poorer quality of life, despite consistently better survival rates (8). Echocardiographic findings show that women have smaller left ventricular (LV) cavities, thicker walls, and greater arterial and ventricular stiffness, manifested by higher E/E′ ratios, lower E′ velocities, and increased diastolic stiffness indices (1, 9).

These sex-specific structural and functional differences may be influenced by hormonal and inflammatory mechanisms. Estrogen deficiency in postmenopausal women promotes systemic inflammation and concentric remodeling, predisposing to diastolic dysfunction and HFpEF (1, 10). Additionally, women rely more on heart rate augmentation to maintain cardiac output under stress, owing to smaller end-diastolic volumes (1).

Analysis of major HFpEF trials demonstrated that older participants were predominantly women with higher comorbidity burden, whereas younger cohorts were mainly obese men, highlighting age- and sex-dependent variations in disease phenotype (11).

#### Mortality vs. symptom burden: paradoxes in outcomes

Risk profiles and pathophysiologic mechanisms differ between sexes, shaping distinct disease trajectories in HF. In essence, obesity, hypertension, and diabetes are more prevalent among women with HFpEF, whereas ischemic heart disease predominates in men (12). Meta-analyses demonstrate that men with HFpEF experience higher all-cause mortality, while hospitalization rates remain comparable between sexes (13). In heart failure with mildly reduced ejection fraction (HFmrEF), men likewise show greater all-cause mortality risk, with no differences in cardiovascular death or HF admissions (13).

Cardiac causes account for a smaller fraction of deaths in HFpEF (≈27%) than in HFrEF (≈65%), suggesting that non-cardiac comorbidities largely drive mortality differences (8, 14). Eventually, across studies, women consistently exhibit better survival than men, especially in non-ischemic HF, though this advantage diminishes in the presence of diabetes (14).

Large pooled analyses, including CHARM-Preserved, I-Preserve, and TOPCAT-Americas, show that women have lower risk of cardiovascular death (HR: 0.70) and combined death/HF hospitalization (HR: 0.80), but similar hospitalization rates (4, 8). The reduced cardiovascular mortality in women is partly explained by a lower incidence of sudden cardiac death (1, 8). Nevertheless, this survival benefit coexists with a paradoxical pattern of poorer health-related quality of life and greater symptom burden among women with HFpEF (4, 8).

#### Temporal evolution: from risk factors to clinical syndromes

A more precise understanding of sex-specific risk factors in HFpEF could refine risk stratification models and enhance women's representation in clinical trials. Beyond conventional contributors such as hypertension, diabetes, and obesity, female-specific determinants—including sex hormone fluctuations, pregnancy-related complications, and other reproductive factors—may partly explain the predominance of HFpEF in women (10).

Comorbidities like obesity, diabetes mellitus, and hypertension promote chronic systemic inflammation—a central mechanism in HFpEF pathogenesis. Subsequently, these conditions are more prevalent in women, they likely contribute to their disproportionate HFpEF risk (10, 15). Obesity, in particular, has emerged as a major driver of HFpEF through mechanisms involving insulin resistance, endothelial dysfunction, and myocardial remodeling (16).

Sex-specific differences in fat distribution further modulate this risk. In particular, premenopausal women tend to accumulate subcutaneous rather than visceral fat, a pattern that reverses with menopause as estrogen levels decline, leading to increased visceral adiposity and proinflammatory states that exacerbate diastolic dysfunction (10, 17, 18).

### The biology of inequality: mechanistic insights

The conceptual basis for understanding sex differences in cardiovascular disease builds on the framework proposed by Arnold, which identifies three main sources of variation:

1. the activational effects of gonadal steroid hormones,
2. the organizational effects of these hormones during development,
3. non-hormonal influences arising from sex chromosome gene expression independent of gonadal function (12).

This model provides a foundation for exploring how both hormonal and genetic mechanisms contribute to the sex-specific pathophysiology observed in HF.

One of the hallmark features of HFpEF in women is increased LV stiffness, accompanied by a smaller LV chamber compared to men (1, 6). Under hemodynamic stress, women exhibit more concentric remodeling, thicker ventricular walls, and a reduced LV end-diastolic volume. Therefore, they depend more on an elevated heart rate to sustain cardiac output due to lower stroke volume. During physical exertion, the greater rise in diastolic stiffness further limits stroke volume augmentation, rendering chronotropic incompetence and exercise intolerance more clinically significant in women (1, 4, 6).

#### Cardiac structure and function: anatomical and hormonal influences—estrogen, myocardial stiffness and fibrosis

Fundamental anatomical and hormonal distinctions contribute to sex-specific patterns of cardiac structure and function. Women typically exhibit smaller LV chambers, lower stroke volumes, higher resting heart rates, and greater systolic and diastolic stiffness compared to men (12). As an illustration, female cardiomyocytes are less prone to apoptosis, and with aging, women tend to develop concentric remodeling and diastolic dysfunction, whereas men more frequently exhibit eccentric remodeling, systolic impairment, and reduced adrenergic responsiveness. Consequently, older men are more likely to develop HFrEF, while women more often progress toward HFpEF (1, 12).

Aging induces ventricular stiffening and extracellular matrix remodeling in both sexes, yet the extent and mechanisms differ. Male hearts accumulate greater myocardial fibrosis than female hearts, partly due to sex-specific regulation of collagen metabolism. Collagen types I and III are lower in young women than in men but are increasing disproportionately with age, suggesting that impaired collagen degradation—rather than enhanced synthesis—drives fibrosis, following distinct temporal trajectories by sex (12, 19).

**Figure 1:**
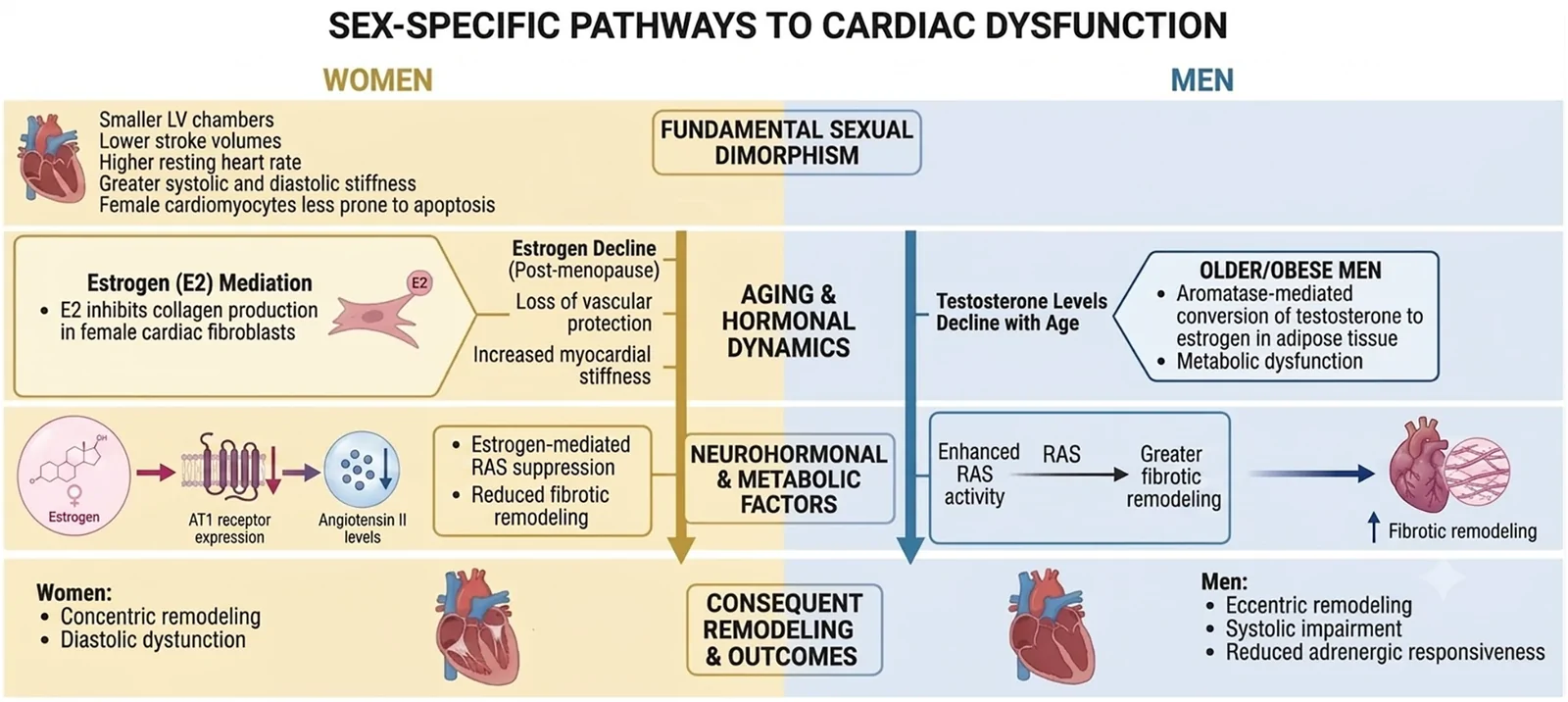
Sex-specific pathways to cardiac dysfunction and heart failure phenotypes. Sex-related differences in cardiac structure, hormonal regulation, and neurohormonal activation contribute to distinct remodeling patterns and heart failure phenotypes. Women are more likely to develop concentric remodeling and diastolic dysfunction, whereas men more frequently exhibit eccentric remodeling and systolic impairment.

Estrogen (17*β*-estradiol, E2) plays a central antifibrotic and cardioprotective role (Figure 1). Experimental data show that E2 inhibits collagen production in cardiac fibroblasts from women but stimulates it in men, highlighting receptor-dependent effects (1, 19). The decline in estrogen levels after menopause correlates with increased myocardial stiffness and loss of vascular protection (12). Estrogen receptors *α* (Er*α*) and *β* (Er*β*) exert opposing influences on collagen synthesis—ER*α* suppresses, while ER*β* promotes expression—through sex-dependent gene regulation (1, 19).

Beyond hormonal modulation, sex-specific neurohormonal activation contributes to these disparities. Enhanced renin–angiotensin system activity in men, alongside estrogen-mediated suppression in women, likely underpins the greater fibrotic remodeling observed in male hearts (1). In parallel, testosterone levels decline with age in both sexes; low androgen levels are linked to impaired myocardial contractility and adverse outcomes, while aromatase-mediated conversion of testosterone to estrogen in adipose tissue contributes to metabolic dysfunction, particularly in older or obese men (1, 12).

#### Titin isoforms, mitochondrial function and metabolic regulation

Sexual dimorphism in HF extends to myocardial energetics and mitochondrial biology. Under pressure overload—an established precursor of hypertrophy and HF—suppression of genes involved in lipid oxidation and energy metabolism is more pronounced in male hearts, whereas females demonstrate better preservation of mitochondrial function and oxidative capacity (1). This metabolic resilience in women is associated with superior calcium handling, lower production of reactive oxygen species, and improved energy efficiency, which together confer relative protection against HF development (1, 6).

In response to hemodynamic stress, female hearts, but not male hearts upregulate genes associated with peroxisome-dependent lipid utilization, reflecting an adaptive mechanism to sustain ATP production when metabolic demands increase (1). Estrogen contributes to this protection by enhancing mitochondrial biogenesis, maintaining electron transport chain integrity, and regulating the expression of mitochondrially encoded genes through direct interaction with mitochondrial DNA (12, 19). These effects reduce ischemia—reperfusion injury and oxidative stress—related myocardial damage.

Moreover, sex hormones modulate arachidonic acid metabolism in divergent ways: under estrogenic influence arachidonic acid is converted into epoxyeicosatrienoic acids with antiarrhythmic and cardioprotective properties, whereas androgens favor hydroxyeicosatetraenoic acid formation, which promotes vasoconstriction and arrhythmogenesis (1, 19). Collectively, these molecular and metabolic mechanisms illustrate how estrogen-mediated preservation of mitochondrial homeostasis and substrate flexibility contributes to the lower incidence and slower progression of HF in women.

#### Inflammation and neurohormonal activation: sex-dimorphic patterns

Inflammation and neurohormonal activation display distinct sex-related trajectories throughout life. While younger men are generally more prone to inflammatory and fibrotic cardiac remodeling, this pattern reverses with aging—older women exhibit greater myocardial inflammation and fibrosis compared to age-matched men (1).

Inflammation represents a central mechanism in the development of HFpEF, with numerous studies linking elevated inflammatory biomarkers to impaired diastolic function and myocardial stiffening (6, 20). The higher prevalence of autoimmune diseases among women, often associated with diastolic dysfunction and endothelial inflammation, exemplifies the connection between immune dysregulation and HFpEF pathogenesis (6).

Women mount stronger innate and adaptive immune responses than men, characterized by greater CD4⁺ and CD8⁺ T-cell activation, elevated circulating cytokines, and enhanced expression of pro-inflammatory genes (6, 20). Although this heightened immune activity may confer protection against infections, it also predisposes to chronic low-grade inflammation, microvascular endothelial dysfunction, and interstitial fibrosis—key pathophysiological hallmarks of HFpEF in women (1, 6, 20).

## The diagnostic dilemma in women

### Clinical presentation: subtle signs, missed signals

Women with HF often present with more subtle or atypical symptoms, contributing to diagnostic uncertainty and delayed recognition. For instance, in a meta-analysis of key HFpEF trials—CHARM-Preserved, I-Preserve, and TOPCAT-Americas—women reported greater symptom burden, higher rates of congestion, and poorer quality of life compared to men, despite similar hospitalization rates and lower cardiovascular mortality (1).

Epidemiological data show that women with HF are typically older, more frequently hypertensive, and less likely to exhibit overt coronary artery disease, while often retaining preserved systolic function (21). These clinical subtleties contribute to diagnostic delay and misinterpretation, frequently leading to under-referral for specialized care (5).

According to Vishram-Nielsen et al. (22), although women may appear more hemodynamically stable, they are equally symptomatic and comorbid as men, underscoring that apparent compensation often masks significant functional impairment. Furthermore, gender-related differences in symptom perception and clinician interpretation may amplify this diagnostic gap (3).

### Sex-related interpretation of biomarkers and imaging

Today, biomarkers have become an integral part of modern HF management, offering insights into underlying pathophysiology, prognosis, and guiding therapeutic decisions. They help clinicians quantify disease severity, monitor response to therapy, and, increasingly, personalize treatment. Despite extensive research, however, few biomarkers have been translated into sex-specific recommendations or incorporated into daily clinical algorithms (23).

A major limitation in current biomarker-based approaches lies in the inadequate understanding of how concentrations differ between healthy women and men and how these differences influence disease interpretation. Many studies overlook the fact that sex differences may arise from both biological and hormonal mechanisms, as well as from differences in body composition, metabolic rate, and renal clearance. Establishing sex-specific reference ranges for key biomarkers, such as natriuretic peptides (NP), cardiac troponins, and inflammatory markers require evaluation within healthy population before being applied to HF cohorts (23, 24).

B-type natriuretic peptide and its inactive amino-terminal fragment (NT-proBNP) are cornerstone biomarkers in diagnosing and stratifying HF, particularly in patients presenting with dyspnea. Circulating concentrations of cardiac NPs are approximately two times higher in women than in men after puberty, largely due to hormonal modulation (24). Estrogen promotes NP gene expression and enhances receptor signaling through the natriuretic peptide receptor-A to receptor-C ratio (NPR-A/NPR-C), while testosterone has a suppressive effect on NP production and secretion. In contrast, some studies suggest that estrogen may also increase neprilysin activity, potentially attenuating this stimulatory effect (24).

In patients with established HF, however, the impact of sex on NP levels becomes less consistent, possibly due to the overriding influence of disease severity and neurohormonal activation. The coexistence of obesity, more common in women with HFpEF, further complicates NP interpretation. Adiposity is associated with lower NP concentrations due to increased clearance and dilutional effects. Notably, recent evidence indicates that the reduction in NT-proBNP levels associated with male sex exceeds that attributable to obesity alone, suggesting that uniform diagnostic thresholds may lead to under-recognition of HF in men (24, 25).

Cardiac troponins (cTnI, cTnT) are sensitive indicators of cardiomyocyte injury and necrosis. Baseline levels are generally higher in men than in women, even in the absence of overt cardiovascular disease (24). This difference reflects, at least in part, greater LV mass and comorbidity burden in men, but may also be influenced by testosterone-mediated increases in myocardial wall stress and apoptosis. In contrast, estrogens exert cardioprotective effects by stabilizing cellular membranes, reducing oxidative stress, and limiting necrosis, resulting in lower troponin concentrations in women. These findings suggest that sex-specific troponin cutoffs could enhance diagnostic accuracy for myocardial injury in HF and acute coronary syndromes (24).

Galectin-3, a *β*-galactoside–binding lectin implicated in fibrosis and inflammation, is expressed by activated macrophages and fibroblasts in the failing myocardium. In healthy populations, plasma galectin-3 levels are slightly higher in women than in men, likely reflecting differences in adipose tissue distribution and systemic inflammation. However, this sex difference becomes inconsistent in HF, suggesting that disease-driven activation of fibrotic pathways overshadows baseline physiological variation (24).

Soluble ST2 (sST2), a marker of myocardial stretch and remodeling, acts as a decoy receptor for interleukin-33, thereby inhibiting its cardioprotective and anti-hypertrophic effects. Men consistently exhibit higher sST2 concentrations than women, both in health and in HF cohorts, regardless of obesity or age. These differences appear only partly explained by sex hormones, and no consistent relationship with body composition has been identified (23, 24). Because sST2 is less influenced by renal function and body mass, it may serve as a robust prognostic marker in both sexes, though sex-specific prognostic thresholds warrant further validation.

#### Inflammatory and metabolic biomarkers

Inflammation plays a crucial role in HFpEF, a phenotype that disproportionately affects women. High-sensitivity C-reactive protein (hsCRP) levels are often higher in women, correlating strongly with body mass index and particularly with subcutaneous fat accumulation. Subcutaneous, rather than visceral, adiposity explains the elevated CRP levels seen in women (25). Moreover, elevated hsCRP has been observed in metabolically at-risk women without established cardiovascular disease, suggesting that it could serve as an early biomarker for cardiovascular risk stratification and HFpEF predisposition (25).

#### Biomarkers of response to therapy

Sex differences are also apparent in biomarker dynamics during treatment. In the PROVE-HF *post hoc* analysis, approximately 30% of HFrEF patients treated with sacubitril/valsartan were women. Following therapy initiation, both sexes demonstrated significant reductions in NT-proBNP; however, women exhibited more consistent and earlier changes. Although women began with lower Kansas City Cardiomyopathy Questionnaire scores—indicating worse baseline health status—they experienced greater early improvements in quality of life than men. Echocardiographic data showed that reverse remodeling occurred earlier in women, though the overall magnitude of improvement was similar across sexes. The study highlights the need for better representation of women in HF trials to elucidate potential sex-based differences in treatment response and biomarker kinetics (26).

#### Imaging biomarkers and structural assessment

Imaging modalities complement circulating biomarkers by providing anatomical and functional insight into sex-specific remodeling. According to Katsuya et al. (27), in patients hospitalized for acute decompensated HFrEF, a larger left ventricular end-diastolic diameter predicted poor outcomes—including cardiac death and readmission—for men, but not for women. This suggests that ventricular remodeling and wall stress may follow different adaptive pathways by sex.

Aligned with, echocardiographic studies summarized by the 2024 Heart Failure Association (HFA) scientific statement show that women with HF more frequently exhibit concentric LV geometry, greater diastolic stiffness, and preserved systolic function—features consistent with the HFpEF phenotype (5). In comparison, men demonstrate more eccentric remodeling, LV dilation, and systolic dysfunction, corresponding to the HFrEF spectrum.

#### Clinical implications

Taken together, these findings demonstrate that biomarker concentrations and imaging parameters are deeply influenced by biological sex, hormonal status, and body composition. Using uniform diagnostic thresholds risks misclassification and underdiagnosis—particularly of women with HFpEF and men with early HFrEF. Future guideline updates should incorporate sex-specific biomarker interpretation, emphasizing differences in NP, troponin, sST2, and CRP behavior, as well as imaging correlates. Integrating sex-aware diagnostic thresholds could substantially improve precision in HF diagnosis, prognosis, and treatment response assessment (3, 23–26).

### Diagnostic delay and mislabeling: HFpEF vs. anxiety, deconditioning

HFrEF and HFpEF represent two pathophysiologically distinct syndromes, differing in etiology, comorbidity profiles, and prognosis (1). Women are disproportionately affected by HFpEF, a condition often underrecognized due to nonspecific presentation, overlapping comorbidities, and the misconception that symptoms such as exertional dyspnea are non-cardiac in origin (2, 5).

Psychological factors, particularly anxiety, may further obscure the diagnostic picture. Although several studies have evaluated the prognostic role of anxiety in HFrEF, its impact in HFpEF remains less clear. Lin et al. demonstrated that while marked differences exist between HFpEF and HFrEF populations in terms of age, sex, comorbidities, and biomarkers, anxiety severity and 18-month outcomes did not differ significantly, suggesting that psychological symptoms may influence perception and reporting of dyspnea rather than disease progression (28).

A frequent diagnostic challenge, particularly in women is unexplained exertional dyspnea in the absence of overt congestion or markedly elevated NP. Such presentations may conceal early or “preclinical” HFpEF, where LV filling pressures rise only under exercise conditions. Up to 20% of HFpEF patients present with normal or borderline NT-proBNP values, leading to delayed recognition or mislabeling as anxiety, deconditioning, or pulmonary disease (29). The reliance on resting echocardiographic findings and standard NP thresholds may, therefore, miss early disease in women whose phenotype is characterized by obesity, hypertension, diabetes, and microvascular inflammation rather than overt systolic dysfunction (5, 6).

Recent work by Berthelot et al. provided mechanistic evidence for these diagnostic limitations. Among patients with unexplained exertional dyspnea and normal LVEF, a subset, all women exhibited higher body mass index, more frequent hypertension and diabetes, and higher (though “normal”) NT-proBNP values compared with controls. Resting echocardiography revealed minimal abnormalities, but exercise testing unmasked pathophysiologic differences: elevated LV filling pressures, reduced peak oxygen uptake (VO₂), and steeper ventilatory efficiency slopes. These findings confirm that static assessments may underestimate early HFpEF, particularly in postmenopausal women with metabolic risk factors (6, 29).

Conversely, deconditioning accounted for a substantial proportion of unexplained dyspnea cases. It often results from inactivity secondary to obesity, diabetes, or chronic pulmonary disease and presents with non-specific cardiopulmonary exercise testing (CPX) findings such as reduced peak VO₂. Distinguishing deconditioning from early HFpEF remains challenging, as both may coexist within a cardiometabolic continuum. Some authors propose that chronic deconditioning, microvascular dysfunction, and inflammation may represent precursor states of HFpEF, particularly in women with sedentary lifestyles and low estrogen levels (1, 6, 10).

CPX and stress echocardiography are crucial tools for unmasking early HFpEF and differentiating it from non-cardiac causes of dyspnea. Early identification and targeted intervention in this phase could reduce future HF hospitalizations and improve the quality of life. However, even with advanced diagnostic tools, a significant subset of patients—most of them women remain unclassified, underscoring the need for refined, sex-sensitive diagnostic algorithms and updated guideline thresholds that integrate hormonal and metabolic factors (1, 2, 5, 29).

## Treatment: equal guidelines, unequal implementation

### Differences in response to guideline-directed medical therapy. Sex-specific pharmacodynamics and therapeutic response

Therapeutic responses in HF show consistent sex-specific variability. Historically, women have been underrepresented in randomized controlled trials of HFrEF, yet *post hoc* and observational analyses indicate that they may derive similar or even greater benefits from standard pharmacologic therapies—such as ACEI, *β*-blockers, and mineralocorticoid receptor antagonists (MRA)—often at lower doses (2, 6, 30). Recent data demonstrate that women achieve equivalent prognostic benefit at approximately half the guideline-recommended doses of ACEI, ARBs, and *β*-blockers, with up to 30% lower composite risk compared to men treated with full-dose therapy (31). These findings highlight the need for optimized, sex-specific dosing strategies in clinical practice, though current HF guidelines remain sex-neutral (5).

Women also experience a higher incidence of adverse drug reactions—particularly hypotension, renal impairment, and cough—which may reduce adherence and worsen long-term outcomes (32). Such differences arise from fundamental physiological and metabolic disparities between sexes. Women's higher body fat increases the volume of distribution of lipophilic agents and prolongs drug exposure, while men's greater lean mass and total body water enhance the distribution of hydrophilic compounds. Additionally, women exhibit lower hepatic CYP1A2 but higher CYP3A4 activity, and sex hormones influence renal clearance and gastrointestinal absorption (2, 3). Together, these factors contribute to sex-specific variations in pharmacodynamics, therapeutic efficacy, and adverse-event profiles.

Major ARB trials—LIFE, ELITE, OPTIMAAL, Val-HeFT, I-Preserve, and CHARM—did not demonstrate significant sex-based differences in efficacy or safety, supporting equal dosing in both sexes (1). As, some studies indicate better adherence and slightly greater efficacy among women with HFrEF receiving ARBs, suggesting subtle sex-related pharmacodynamic differences (2, 30).

Regarding ARNIs, the PARADIGM-HF trial confirmed that sacubitril/valsartan was superior to enalapril in reducing mortality and hospitalization for both sexes, with a slightly greater cardiovascular mortality reduction in men. Conversely, the PARAGON-HF trial demonstrated a pronounced benefit in women, who represented over half the cohort, suggesting that HFpEF in women may respond particularly well to neprilysin inhibition (1, 2, 6).

For ACEI, sex-based pharmacokinetic and hormonal influences modulate both efficacy and tolerability. Estrogen and progesterone reduce ACE activity—particularly during the follicular phase—while androgens enhance renin-angiotensin-aldosterone system activation (31). Consequently, postmenopausal women may respond better to ACEI than to ARBs, possibly due to increased angiotensin II receptor expression (1). Early trials (CONSENSUS I, SAVE, SOLVD) showed smaller mortality reductions in women, but later studies (AIRE, HOPE and BIOSTAT-HF) confirmed comparable efficacy, with optimal outcomes achieved at lower doses in women (1, 30, 31). Thus, adverse reactions—especially dry cough—remain more frequent in women (32).

Similarly, *β*-blockers remain a cornerstone of HFrEF therapy, but current guidelines lack sex-specific dosing recommendations (5). Women typically have higher plasma concentrations of metoprolol and propranolol due to slower clearance and smaller distribution volume, implying that equivalent systemic exposure can be achieved with approximately half the male dose (2, 31). Hormonal influences further modulate *β*-adrenergic signaling: estrogen and progesterone attenuate *β*₁-receptor expression, while women demonstrate higher sympathetic tone and enhanced *β*₂-receptor sensitivity (2, 6). Although meta-analyses by Danielson and Kotecha found no significant sex-based differences in overall efficacy, observational data by Santema et al. indicated that women treated with lower doses of *β*-blockers and ACEI/ARB experienced fewer adverse events and similar outcomes compared to men (30, 31).

MRA—spironolactone, eplerenone, finerenone—show largely comparable benefits across sexes in large-scale trials such as RALES, EMPHASIS-HF, and TOPCAT (1, 2). However, the TOPCAT *post hoc* analysis revealed that spironolactone reduced mortality in women with HFpEF (HR: 0.66, *p* = 0.01), but not in men (*p* for interaction = 0.02), suggesting a potential survival advantage for women (33).

Collectively, these findings support the rationale for sex-tailored pharmacologic strategies. Biological and hormonal differences influence both efficacy and safety, emphasizing the need for dose optimization in women to improve tolerability and adherence while maintaining equivalent prognostic benefit (5, 6, 30–32).

### Device therapy and transplant: sex-based access inequalities

Implantable cardioverter-defibrillator (ICD) therapy confers comparable survival benefits in women and men with both ischemic and non-ischemic cardiomyopathy. Nonetheless, women remain markedly underrepresented, accounting for only 10%–25% of participants in clinical trials and registries (1, 34, 35). Despite similar indications, they are significantly less likely to be referred for ICD implantation, particularly for primary prevention (3, 5). Registry data consistently show that women present more often with non-ischemic cardiomyopathy, fewer comorbidities, and higher procedural complication rates, yet receive fewer appropriate ICD shocks—differences attributed primarily to selection and referral biases rather than biological divergence (34, 35).

Observational analyses from Medicare and the GWTG-HF program confirm persistent underuse of ICDs among eligible women, even after adjusting for age, ejection fraction, and comorbidities. The Belgian nationwide registry [Ingelaere et al. (34),] reported a 7:1 male-to-female implantation ratio in ischemic cardiomyopathy and 2.6:1 in non-ischemic forms, far exceeding disease prevalence ratios—evidence of systematic undertreatment (34). Female recipients were typically younger, with fewer comorbidities, and more often diagnosed with arrhythmogenic or dilated cardiomyopathy, yet sex was not an independent predictor of survival (35).

Educational and system-level interventions, such as the IMPROVE-HF and GWTG-HF programs, demonstrated moderate success in closing this gap by integrating clinician feedback, decision-support tools, and patient education (1, 34, 35). Nevertheless, access inequalities persist—particularly in primary prevention—highlighting the need for sex-sensitive referral criteria, balanced trial enrollment, and equitable access to device-based therapies and transplantation for women (1, 3, 5, 34, 35).

## Research design and clinical trials: where are the women?

Despite comparable prevalence of HF between sexes, women remain markedly underrepresented in cardiovascular clinical trials. A comprehensive analysis of 740 cardiovascular studies found that women constituted only 38.2% of participants, while a meta-analysis by Danielson et al. revealed that merely 23.1% of subjects in HF pharmacotherapy trials were female (31). These findings underscore a long-standing imbalance that undermines the generalizability of current evidence to female populations.

Several systemic barriers contribute to this underrepresentation. Historical exclusion criteria, advanced age at disease onset in women, and comorbidity patterns (such as hypertension and diabetes rather than ischemic heart disease) often limit eligibility for enrollment (3). Sociocultural and logistical factors—including caregiving responsibilities and lower referral rates—further reduce female participation. Importantly, even when women are included, trial analyses frequently omit sex-stratified results, perpetuating knowledge gaps in treatment response and safety (1, 3, 5).

Recent scientific statements by the HFA and other expert groups emphasize the urgent need for sex-balanced recruitment, transparent sex-specific reporting, and outcomes extending beyond mortality—such as symptom burden, quality of life, and adverse drug reactions (2, 5, 31). Addressing these disparities is essential to advance equitable and evidence-based management of HF in both women and men.

## Clinical practice guidelines: neutral or neglectful?

Contemporary ESC/ACC/AHA HF guidelines remain largely sex-neutral in recommendations and dosing targets, despite mounting evidence of sex-specific biology, pharmacokinetics, safety profiles, and therapeutic response (1–3, 5, 31). This neutrality contributes to systematic blind spots—particularly in HFpEF—where women are overrepresented, present differently, and often experience greater symptom burden but better survival (1, 4, 8).

### Do current ESC/ACC/AHA guidelines integrate sex-specific evidence?

Guidelines endorse uniform GDMT targets and device indications with minimal sex-stratified guidance (2, 5). Yet, multiple sources document that women may achieve equivalent prognostic benefit at lower doses for ACEI/ARB/*β*-blockers (30, 31), report more frequent adverse drug reactions (32), and show heterogeneous responses to MRA and ARNI across the EF spectrum (1, 33). For diagnosis, guideline pathways center on NP and resting imaging, although sex affects biomarker baselines and phenotypic expression (23, 24). Overall, current texts summarize data but seldom operationalize it into sex-specific cut-offs, dose bands, or pathways (2, 5).

### The case for sex-sensitive diagnostic and therapeutic algorithms

Sex influences HF risk factors (including female-specific risks), pathophysiology (inflammation–metabolic axis, fibrosis, ventricular–arterial stiffening), biomarker levels, and treatment response (1, 10, 20, 23, 24). Women more often have HFpEF with greater symptom burden and lower sudden death risk; men more often have HFrEF with higher ischemic burden (4, 8, 14). Sex-sensitive algorithms would therefore:

1. adapt diagnostic thresholds/steps for biomarkers and exertional testing;
2. allow dose-optimization strategies acknowledging differential exposure and ADR propensities;
3. incorporate outcomes beyond mortality (quality of life, function) that track women's disease burden (5, 23, 31, 32).

### Missed opportunities in current policy

Three gaps recur across guidance and implementation: underrepresentation of women in the trials that inform guidelines; lack of mandated sex-stratified analyses/reporting; and limited integration of sex-specific biomarker interpretation, dosing, and device access guardrails (3, 5, 31, 34, 35). These gaps propagate downstream inequities—e.g., lower referral and implant rates for ICDs in eligible women despite similar survival benefit when treated (34, 35).

## Future directions and implications for clinical practice and research. Importance of integrating sex and gender in diagnostic and therapeutic algorithms

**Diagnosis—**Adopt sex-aware interpretation of biomarkers (NPs, troponin, galectin-3, sST2) with consideration of baseline differences, adiposity patterns, and HF phenotype; embed exercise-based evaluations earlier for dyspnea with normal/low NP, where women with incipient HFpEF are frequently missed (24, 29, 36, 37).

**Therapy—**Implement dose-finding/titration pathways that target equal benefit rather than equal dose, reflecting women's higher exposure and ADR risk at the same nominal dose (30–32). Consider phenotype-specific signals (e.g., spironolactone in HFpEF subgroups; differential ARNI effects across EF and sex) while acknowledging evidentiary limits (1, 26, 33, 38).

**Devices—**Build referral checklists and audit/feedback loops to counter lower ICD use in eligible women; track procedural complications and optimize peri-procedural care where female sex predicts higher complication risk (5, 34, 35).

**Knowledge gaps—**Priority areas include: validated sex-specific biomarker cut-offs; prospective dose-ranging by sex; mechanistic links among inflammation–metabolism–fibrosis and female-predominant HFpEF; and outcomes beyond mortality (1, 10, 20, 23, 39).

## Conclusions: bridging the gap, rebalancing the field

HF represents a heterogeneous syndrome profoundly influenced by sex and gender across its entire continuum—from risk factors and pathophysiology to diagnosis, treatment, and outcomes. The traditional “one-size-fits-all” approach, derived largely from male-dominated evidence, fails to capture the biological and clinical nuances that characterize HF in women.

Women are more frequently affected by HFpEF, driven by mechanisms that include hormonal changes, microvascular dysfunction, inflammation, and metabolic dysregulation. Despite better survival, they endure a higher symptom burden and poorer quality of life, illustrating the paradox between longevity and well-being. In contrast, men predominate in HFrEF, with more ischemic etiologies and a greater risk of sudden cardiac death. These diverging trajectories underscore the need for a sex-informed understanding of cardiac aging, remodeling, and neurohormonal regulation.

Diagnostic pathways remain biased toward male phenotypes, leading to delayed recognition or misclassification of HF in women—particularly in early or atypical HFpEF presentations. Sex-based differences in biomarker profiles, imaging patterns, and symptom perception demand tailored diagnostic thresholds and evaluation strategies. Similarly, therapeutic responses to GDMT reveal significant pharmacokinetic and pharmacodynamic disparities, suggesting that equivalent benefit may be achieved at lower doses in women. Yet, despite equal potential for benefit, device implantation and advanced therapies continue to reflect gendered inequities in access and referral.

Progress will depend on a deliberate shift toward equity in both research and practice. Clinical trials must ensure balanced enrollment, mandatory sex-stratified analyses, and endpoints reflecting quality of life as well as mortality. Guidelines must evolve from sex-neutral to sex-informed, integrating biological and sociocultural dimensions of cardiovascular disease.

To conclude, reframing HF through a gender-sensitive lens is not a matter of fairness but of precision. Recognizing and operationalizing sex and gender differences in HF care will enhance diagnostic accuracy, optimize therapy, and bring the field closer to truly personalized—and equitable—cardiovascular medicine.
