# Correction: Gender disparities in heart failure diagnosis and management: under the radar

**DOI:** 10.3389/fcvm.2026.1973162

**Published:** 2026-09-03

**Authors:** Irina Cabac-Pogorevici, Iuliana Creangă, Inessa Jitari, Dmitri Savca, Valentin Avram, Alexandru Corlăteanu, Valeriu Revenco

**Affiliations:** 1 Cardiology Laboratory, “Nicolae Testemițanu” State University of Medicine and Pharmacy, Chișinău, Republic of Moldova; 2 Discipline of Cardiology, “Nicolae Testemițanu” State University of Medicine and Pharmacy, Chișinău, Republic of Moldova; 3 Discipline of Pneumology and Allergology, “Nicolae Testemițanu” State University of Medicine and Pharmacy, Chișinău, Republic of Moldova

**Keywords:** biomarkers, gender disparities, guideline-directed medical therapy, heart failure, sex differences

The funder National Agency for Research and Development of the Republic of Moldova was erroneously omitted.

The original version of this article has been updated.

