# Correction: Gender disparities in heart failure diagnosis and management: under the radar

**DOI:** 10.3389/fcvm.2026.1998092

**Published:** 2026-09-23

**Authors:** Irina Cabac-Pogorevici, Iuliana Creangă, Inessa Jitari, Dmitri Savca, Valentin Avram, Alexandru Corlăteanu, Valeriu Revenco

**Affiliations:** 1 Cardiology Laboratory, “Nicolae Testemițanu” State University of Medicine and Pharmacy, Chișinău, Republic of Moldova; 2 Discipline of Cardiology, “Nicolae Testemițanu” State University of Medicine and Pharmacy, Chișinău, Republic of Moldova; 3 Discipline of Pneumology and Allergology, “Nicolae Testemițanu” State University of Medicine and Pharmacy, Chișinău, Republic of Moldova

**Keywords:** biomarkers, gender disparities, guideline-directed medical therapy, heart failure, sex differences

The details for the funder National Agency for Research and Development of the Republic of Moldova was erroneously omitted.

The correct Funding statement reads: The author(s) declared that financial support was received for this work and/or its publication. This publication was funded by the National Agency for Research and Development (NARD) through the project “Multidimensional Approach to Chronic Heart Failure for the Development of a Personalized Assessment Matrix” (grant No. 26.00208.8007.01/PD), within the framework of the “Postdoctoral Programs “and Nicolae Testemițanu State University of Medicine and Pharmacy from the Republic of Moldova.

The original version of this article has been updated.

